# Increased hexokinase-2 as a novel biomarker for the diagnosis and correlating with disease severity in rheumatoid arthritis

**DOI:** 10.1097/MD.0000000000026504

**Published:** 2021-06-25

**Authors:** Kai-Long Zhou, Zhen-Hua Zhu, Ju-Pu Zhou, Jia-Ju Zhao, Yong Zhang, Bo Jiang

**Affiliations:** Department of Orthopaedics, The Second Affiliated Hospital of Soochow University, Suzhou, Jiangsu, China.

**Keywords:** biomarker, glucose metabolism, hexokinases-2, rheumatoid arthritis

## Abstract

Abnormal glucose metabolism brings out joint inflammation and destruction in rheumatoid arthritis (RA). The aim of this study was to evaluate the potential of circulating hexokinase-2 (HK2) in peripheral blood mononuclear cells (PBMCs) of rheumatoid arthritis (RA) patients.

PBMCs were obtained from patients with RA or osteoarthritis (OA) and healthy controls (HCs). The expression of HK2 was assessed by quantitative reverse transcription-polymerase chain reaction (qRT-PCR). The C-reactive protein (CRP) level, erythrocyte sedimentation rate (ESR), Calprotectin, rheumatoid factor (RF), anti-cyclic citrullinated peptides (anti-CCP) antibody level and 28-joint Disease Activity Score (DAS28), Clinical Disease Activity Index (CDAI) and Simplified Disease Activity Index (SDAI) were measured. Spearman^'^s analysis was performed to determine the association between the level of HK2 and clinical characteristics. A receiver operating characteristic (ROC) curve was employed to evaluate the diagnostic value of HK2 in PBMCs. Logistic regression was used to identify risk factors. Sixty-five RA patients, 35 OA patients, and 40 HCs were included in the study.

HK2 was upregulated in RA and OA patients compared with that in HCs (*P* < .05). The area under the ROC of HK2 for diagnosing RA and OA was 0.808 and 0.640, respectively. In addition, HK2 levels were increased in active RA compared with those in remittent RA (*P* = .03). Furthermore, HK2 correlated positively with the DAS28-ESR (*P* < .001), CDAI (*P* = .02) and SDAI scores (*P* = .02). Moreover, HK2 was independently associated with an increased risk of disease activity (DAS28-ESR>3.2, *P* = .02; CDAI score>10, *P* = .03; SDAI score>11, *P* = .04). Additionally, HK2 positivity was more frequently detected in patients treated with biologic disease-modifying antirheumatic drugs (bDMARDs) than in those not treated with bDMARDs.

HK2 levels in PBMCs can be considered an ideal biomarker for diagnosing RA and involved in disease activity in RA. Dysregulation of HK2 may participate in the molecular mechanism of RA and could be an attractive selective metabolic target for RA treatment.

## 1 Introduction

Rheumatoid arthritis (RA) is a chronic autoimmune disease that is characterized by persistent synovitis, the development of joint damage and bone destruction, leading to a high rate of disability and quality of life deterioration.^[1]^ Worldwide, more than 0.5% 1% of the global population suffers from RA.^[2]^ Early diagnosis, regular monitoring and proper treatment may suppress severe clinical manifestations in RA. Traditional serological markers, such as C-reactive protein (CRP),^[3]^ erythrocyte sediment rate (ESR),^[4]^ Calprotectin,^[5]^ rheumatoid factor (RF),^[6]^ and anti-cyclic citrullinated peptide antibody (anti-CCP),^[7]^ is commonly included as a surrogate marker of disease activity or applied as a valuable diagnostic biomarker in RA. However, current methods of diagnosis and evaluation of disease activity demonstrate various insufficiencies in the diagnosis of RA, suggesting the urgency to investigate a novel candidate that may improve diagnosis and monitoring.

Osteoarthritis(OA) is a musculoskeletal disease which is characterized by joint pain, tenderness, stiffness and limitation of movement and variable degrees of local inflammation.^[8]^ Recent studies have proven that glucose metabolism plays a vital role in the pathogenesis of RA and OA.^[9,10]^ Further study showed that glucose metabolism is altered in the synovial fluid and synovium of RA and that the switch to glycolysis apparently supports the high proliferation and aggressive phenotype of RA.^[11,12]^ Furthermore, macrophages and dendritic cells (DCs), two other immune contributors to RA and OA, also experience a metabolic shift from oxidative phosphorylation to glycolysis, therefore confirming a proinflammatory phenotype.^[13]^

Glucose metabolism is regulated by hexokinases (HKs), fructose 2,6-bisphosphate and membrane glucose transporters (GLUTs).^[14,15]^ HK catalysis is the first step in glucose metabolism, and then glucose is phosphorylated by HKs to produce glucose-6-phosphate via GLUTs on the plasma membrane. There are four isoforms, HK-1, HK-2, HK-3, and HK-4, in various tissues. Among them, HK2, which has a high affinity for glucose and contains two catalytic domains, is the main induced isoform.^[16]^ In addition, increased HK2 in fibroblast-like synoviocytes (FLSs) was demonstrated to have a relationship with the aggressive phenotype of RA.^[11]^ However, the role of HK2 in peripheral blood mononuclear cells (PBMCs) of RA has recently been uncovered, and the mechanisms responsible for glucose metabolism in peripheral blood need to be explored in more detail.

In the present study, we tested the hypothesis that the PBMCs levels of HK2, reflecting inflammation, may be involved in RA. Our study detected the relative expression levels of HK2 among RA, OA patients and healthy individuals (HCs) through real-time quantitative PCR method. Roc curve analysis was performed to assess the performance of HK2 in the diagnosis of RA, OA and HCs compared with that of CRP, ESR, calprotectin. The association of circulating HK2 expression with the risk and disease severity of RA were determined. Taken together, our data suggest that HK2 may be used as a novel biomarker for RA diagnosis and involved in disease activity and could play a role in RA.

## 2 Materials and methods

### 2.1 Patients

This horizontal observational study enrolled 65 RA patients (according to the 2010 American College of Rheumatology (ACR) classification criteria)^[17]^ and matched control groups (35 OA patients and 40 HCs). The participants were recruited between January 2018 and March 2020. All RA patients were assessed for tenderness and swollen joints by experienced study nurses. Disease activity of RA was assessed by the 28-joint Disease Activity Score (DAS28), using tender joint count (TJC) and swollen joint count (SJC), erythrocyte sedimentation rate (ESR) and the patient's and physician's global assessment of activity on the visual analogue scale (VAS) or Clinical Disease Activity Index (CDAI) and Simplified Disease Activity Index (SDAI).^[18]^ Details on demographic data, disease duration, autoantibody status, and disease-modifying antirheumatic drug (DMARD) therapy were collected. RA patients were grouped according to the disease activity states of remission and active stage based on the DAS28-ESR value. Remission was defined as DAS28-ESR <2.6; low disease activity was defined as DAS28-ESR ≤ 3.2; moderate and high disease activity was defined as DAS28-ESR > 3.2.^[19]^ All of selected RA patients met the following criteria:

1. age of participant over 18 years;
2. RA duration longer than 3 months.

The exclusion criteria were set as follows:

1. occurrence of opportunistic infections in the last two months;
2. occurrence of acute infections in a last month;
3. lung, liver or kidney insufficiency;
4. coexistence of diabetes mellitus;
5. Malignant tumor and anti-tumor therapy during last 5 years;
6. HIV infection;
7. pregnancy
8. other autoimmune disorders including systemic lupus erythematosus, Hashimoto's thyroiditis, and so on.^[20]^

Fasting peripheral venous specimen were collected on the day of clinical examination.

### 2.2 Ethical statement

All protocols involving human subjects were approved by the Ethics Committee of the Second Affiliated Hospital of Soochow University (Jiangsu, China). Informed consent was obtained from all the participants.

### 2.3 Serological evaluations

Data from laboratory tests, such as the levels of C-reactive protein (CRP), erythrocyte sediment rate (ESR), Calprotectin, rheumatoid factor (RF), and anti-cyclic citrullinated peptide antibody (anti-CCP), were measured on the day of clinical examination. CRP was measured using turbidimetry (Boyuan Medical Technology Co., Ltd, Shanghai, China). ESR was measured on a PRECIL-XC-40B instrument (Mindray, China). Calprotectin was measured by a commercially available calprotectin enzyme-linked immunosorbent assay (ELISA) kit according to the manufacturer's protocol (Sun Biomedical Technology Co., Ltd, Beijing, China). RF was tested by nephelometric method (Beckman Coulter, Inc., USA). Positive RF was defined as RF>20.0IU/ml. Anti-CCP antibodies were analyzed using chemiluminescence assay (Abbott GmbH & Co.KG, Wiesbaden, Germany). Positive anti-CCP was defined as anti-CCP>5.0U/ml.

### 2.4 Extraction of total RNA from PBMCs

PBMCs were separated after blood sample collection from each subject according to the manufacturer^'^s protocol (GE Healthcare, Uppsala, Sweden). Three millilitres of blood diluted in an equal volume of saline solution was layered onto 6 mL of Ficoll-Paque PLUS. After centrifugation at 400 × g for 30 min at room temperature, the interlayer was collected by washing twice with saline solution. The precipitate was collected by centrifuging at 90 × g for 15 min. Then, PBMCs were frozen at –80° for further testing HK2.

### 2.5 Reverse transcription-quantitative polymerase chain reaction (RT-qPCR)

TRIzol reagent (Invitrogen, Carlsbad CA, United States) was used for RNA extraction from the PBMCs. RNA quality was examined by gel electrophoresis, and the RNA samples were subjected to RT (Real-time) for the synthesis of cDNA using a PrimerScript Real-time Reagent Kit (TaKaRa, Shiga, Japan). Then, the expression of HK2 was quantitated with TB Green^TM^ Premix Ex Taq^TM^ II (TaKaRa, Shiga, Japan) according to the manufacturer's protocols and a LightCycler 480II real-time PCR system (Roche, Rotkreuz, Switzerland). β-actin was used as an internal reference. The primer sequences are presented in Table 1. In total, the thermocycling conditions for PCR were 50 s at 95°, followed by 40 cycles of denaturation at 95° for 12 s and annealing and extension at 60° for 30 s. The 2^−ΔCt^ method was employed to process the data.

**Table 1:** Sequences of primers used for quantitative reverse transcription–polymerase chain reaction.

Name	Primer Sequence
β-actin	F: 5’-GTGGCCGAGGACTTTGATTG 3’ R: 5’-CCTGTAACAACGCATCTCATATT-3’
HK2	F: 5’- GGGCATCTTGAAACAAG -3’ R: 5’- GGTCTCAAGCCCTAAG -3’

### 2.6 Statistical analysis

The data are reported as the mean ± standard deviation (SD) or median (interquartile range). The *t*-test or Mann–Whitney *U*-test was used to compare continuous variables. One-way analysis of variance (ANOVA), Kruskal–Wallis test by ranks, or χ^2^ test was used for multiple comparisons. Correlations were calculated by Spearman's analysis in different groups. Receiver operating characteristic (ROC) curve analysis was applied to evaluate the clinical diagnostic value. The optimal cut-off point of HK2 is selected according to the maximum of the Youden index, and the sensitivity and specificity were identified. A *P* value less than .05 was considered significant. The statistical analyses were conducted using GraphPad Prism 7.04 (GraphPad Software, San Diego, CA, United States) and the SPSS 19.0 (SPSS Inc., IBM, United States) for Windows package.

## 3 Results

### 3.1 Patient characteristics

For the 140 enrolled patients (65 RA, 35 OA and 40 HCs) in the study, Table 2 shows the clinical characteristics at baseline. In total, there were 65 RA patients, 58.46% female, with a mean age of 54.72 ± 11.43 years and mean disease duration of 10.91 ± 4.91 years, as well as 35 OA and 40 HCs used as controls, with a mean age of 55.05 ± 10.73 years for OA and 50.33 ± 9.13 years for HCs, 51.43% female for OA and 55.00% female for HCs. Comparison of RA patients with OA patients and HCs showed no differences between the baseline characteristics, such as age and sex. RF and anti-CCP antibody positivity were found in 56.92% and 53.85% of RA patients, respectively. Serum CRP, ESR, calprotectin levels had statistical difference among RA, OA and HCs groups (*P* < .001). In RA, the median baseline DAS28-ESR value was 3.74, the CDAI was 15.78, and the SDAI was 13.85, indicating that the average patient experienced moderate disease activity. Among them, the most commonly used medication was conventional synthetic disease-modifying antirheumatic drugs (csDMARDs). Twelve patients were treated with biologic DMARDs (bDMARDs).

**Table 2:** Baseline characteristics of the patients with Rheumatoid arthritis (RA), Osteoarthritis (OA) and heathy controls (HCs).

Features	RA (n = 65)	OA (n = 35)	HCs (n = 40)	*P*
Mean age (yr)	54.72 ± 11.43	55.05 ± 10.73	50.33 ± 9.13	.87^∗^
Gender (Male/ Female)	27/38	17/18	18/22	.64^∗^
Disease duration (yr)	10.91 ± 4.91	9.96 ± 3.95	–	.59^†^
CRP, mg/dl	17.5 ± 11.1	12.5 ± 10.7	7.7 ± 4.6	<.001^∗^
ESR, (mm/h)	26.0 ± 15.2	18.1 ± 14.0	7.0 ± 5.7	<.001^∗^
Calprotectin, μg/mL	10.01 (5.01–14.44)	7.72 (4.69–12.68)	4.44 (2.62–7.34)	<.001^∗^
RF, no. positive (%)	37/65 (56.92%)	–	–	
Anti-CCP positivity, n (%)	35/65 (53.85%)	–	–	
TJC [n]; median (range)	7.0 (0.0–28.0)	–	–	
SJC [n]; median (range)	4.0 (0.0–27.0)	–	–	
DAS28-ESR score	3.74 (2.52-6.07)	–	–	
CDAI score	15.78 (2.94-34.37)	–	–	
SDAI	13.85 (4.64–39.65)	–	–	
csDMARDs, n (%)	54/65 (83.08%)		–	
bDMARDs, n (%)	11/65 (16.92%)		–	

Values are shown as mean ± standard deviation (SD) or median (interquartile range).

∗ Comparison among three groups, determined by One-way analysis of variance (ANOVA), Kruskal–Wallis test by ranks, or χ^2^ test.

† Comparison between RA and OA groups, determined by Mann–Whitney-*U*-test. RA = Rheumatoid arthritis; OA = Osteoarthritis; HCs = heathy controls; CRP = C-reactive protein; ESR = erythrocyte sedimentation rate; RF = rheumatoid factor; Anti-CCP = anti-cyclic citrullinated peptides; TJC = Tender joint count; SJC swollen joint count; DAS28-ESR = Disease Activity Score for 28 joints with erythrocyte sedimentation rate; CDAI = Clinical Disease Activity Index; SDAI = Simplified Disease Activity Index; csDMARDs = conventional synthetic disease-modifying antirheumatic drugs; bDMARDs = biologic disease-modifying antirheumatic drugs.

### 3.2 HK2 expression in PBMCs from RA and OA patients

**Figure 1:**
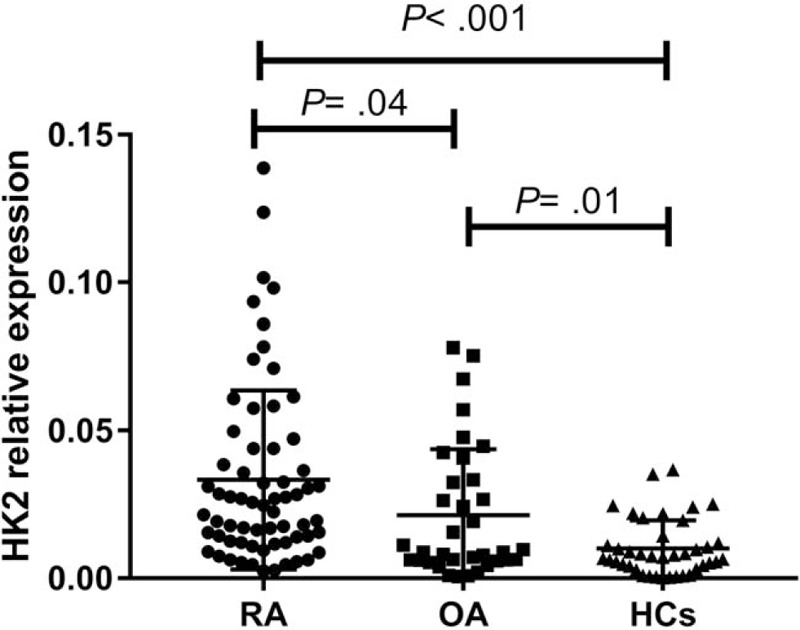
Hexokinase 2 (HK2) relative expression in patients with Rheumatoid arthritis (RA), Osteoarthritis (OA) and healthy controls (HCs). HK2 was increased in RA and OA patients compared with HCs. A significant difference in HK2 was detected between RA and HCs. *P* < .05 was considered statistically significant. HK2: Hexokinase 2; RA: Rheumatoid arthritis; OA: Osteoarthritis; HCs: heathy controls.

**Figure 2:**
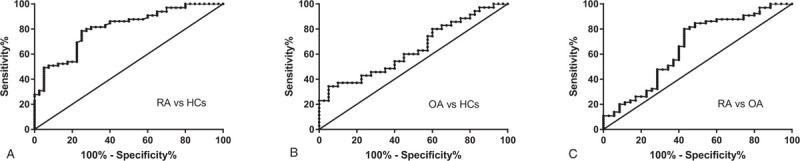
Receiver operating characteristic curves of Hexokinase 2 (HK2) for prediction of Rheumatoid arthritis (RA) and Osteoarthritis (OA) patients. A: HK2 was able to differentiate RA from HCs; B: HK2 could differentiate OA from HCs; C: HK2 was able to differentiate RA from OA. HK2: Hexokinase 2; RA: Rheumatoid arthritis; OA: Osteoarthritis; HCs: heathy controls.

The expression of HK2 in PBMCs was increased in RA (0.033 ± 0.004) compared with that in HCs (0.010 ± 0.002, *P* < 0.001) and OA patients (0.021 ± 0.004, *P* = .04). The expression level of HK2 was higher in PBMCs from the OA group than in those from the HCs group (*P* = .01) **(**Figure 1**)**. The diagnostic value of HK2 in differentiating RA patients from HCs [area under the curve (AUC) = 0.808, 95% confidence interval (CI): 0.725–0.891] and OA patients (AUC = 0.655, 95% CI: 0.536–0.773) was examined **(**Figure 2A and C, Table 3). Overall, HK2 may be considered a predictive factor in differentiating RA or OA from HCs (Figure 2B, Table 3). The level of HK2 was considered positive at a cut-off value > .0113 for RA (Table 3).

**Table 3:** Receiver-operating characteristic analysis of Hexokinase 2 (HK2) in peripheral blood mononuclear cells from patients with Rheumatoid arthritis (RA) and Osteoarthritis (OA).

Group	AUC (95%CI)	*P*-value	Cut off value	Sensitivity (95%CI)	Specificity (95%CI)
RA vs HCs	0.808 (0.725–0.891)	<.001	0.0113	80.00% (68.23%–89.90%)	72.50% (56.11%-85.40%)
OA vs HCs	0.640 (0.514–0.766)	.04	0.0097	48.57% (31.38%–66.01%)	65.00% (48.32%-79.37%)
RA vs OA	0.655 (0.536–0.773)	.01	0.0193	55.38% (42.53%–67.73%)	62.86% (44.92%- 78.53%)

RA = Rheumatoid arthritis; OA = Osteoarthritis; HCs = heathy controls; HK2 = Hexokinase 2; AUC = Area under the curve; CI = confidence interval.

Meanwhile, the diagnostic value was discovered for CRP, ESR and calprotectin in differentiating RA patients from HCs (CRP: AUC = 0.765, 95%CI:0.636–0.823; ESR: AUC = 0.708, 95%CI:0.624–0.802; calprotectin: AUC = 0.812, 95%CI: 0.692–0.870) **(**Table 4**)**. However, CRP, ESR and calprotectin failed as a predictive factor in differentiating RA from OA patients (CRP: AUC = 0.565, 95%CI: 0.401–0.633; ESR: AUC = 0.543, 95%CI: 0.416–0.692; calprotectin: AUC = 0.570, 95%CI: 0.408–0.641) **(**Table 5**)**.

**Table 4:** Receiver-operating characteristic analysis of C-reactive protein (CRP), erythrocyte sediment rate (ESR) and calprotectin from patients with Rheumatoid arthritis (RA) and healthy individuals (HCs).

Parameter	CRP	ESR	Calprotectin
AUC (95%CI)	0.765 (0.636–0.823)	0.708 (0.624–0.802)	0.812 (0.692–0.870)
*P*-value	<.001	.001	<.001
Cut off value	7.5	8.5	5.34
Sensitivity	72.31% (59.81%–82.69%)	63.08% (50.2%–74.72%)	78.46% (66.51%–87.69%)
Specificity	62.50% (45.8%–77.27%)	60.50% (43.33%–75.14%)	75.00% (58.8%–87.31%)

RA = Rheumatoid arthritis; HCs = heathy controls; AUC = Area under the curve; CI = confidence interval.

**Table 5:** Receiver-operating characteristic analysis of C-reactive protein (CRP), erythrocyte sediment rate (ESR) and calprotectin from patients with Rheumatoid arthritis (RA) and Osteoarthritis (OA).

Parameter	CRP	ESR	calprotectin
AUC (95%CI)	0.565 (0.401–0.633)	0.543 (0.416–0.692)	0.570 (0.408–0.641)
*P*-value	.43	.38	.34
Cut off value	–	–	–
Sensitivity	–	–	–
Specificity	–	–	–

RA = Rheumatoid arthritis; OA = Osteoarthritis; AUC = Area under the curve; CI = confidence; CRP = C-reactive protein; ESR = erythrocyte sediment rate.

### 3.3 Correlations of HK2 expression with disease activity in RA

**Figure 3:**
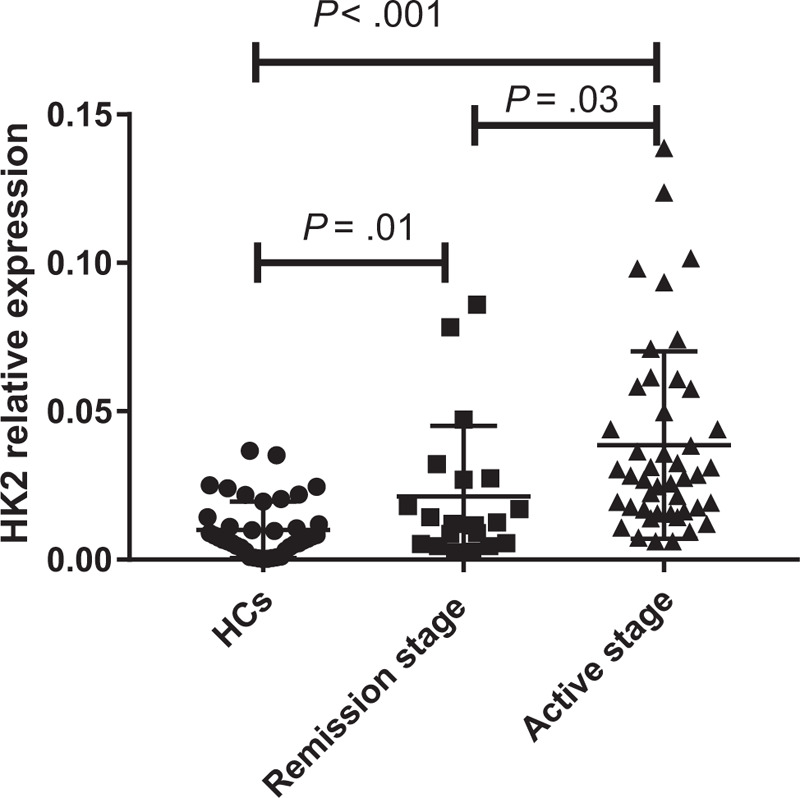
Expression of Hexokinase 2 (HK2) in peripheral blood mononuclear cells from Rheumatoid arthritis (RA) at different stages. *P* < .05 was considered statistically significant. HK2: Hexokinase 2; RA: Rheumatoid Arthritis, HCs: Healthy controls.

In the entire cohort, using DAS28-ESR criteria, 70.77% (46/65) of patients were classified as having an active disease stage (DAS28-ESR≥2.6). Nevertheless, 29.23% (19/65) exhibited clinical remission (DAS28-ESR<2.6). The expression level of HK2 was markedly increased, with values 3.819-fold higher, in PBMCs from active RA (*P* < .001). However, expression was only 2.110-fold higher in RA patients who were in remission (*P* = .01), and HK2 levels in active RA were higher than those in remittent RA (*P* = .03) **(**Figure 3**)**. The diagnostic value of HK2 in differentiating active RA from remittent RA [AUC = 0.737, 95% CI: 0.594–0.880] was examined **(**Table 6**)**.

**Table 6:** Receiver-operating characteristic analysis of rheumatoid factor (RF) and anti-cyclic citrullinated peptide antibody (anti-CCP) from active Rheumatoid arthritis (RA) and RA in remission.

Parameter	HK2	RF	anti-CCP
AUC (95%CI)	0.737 (0.594–0.880)	0.589 (0.443–0.733)	0.618 (0.475–0.761)
*P*-value	.03	.24	.12
Cut off value	0.0186	–	–
Sensitivity	68.89% (53.35%–81.83%)	–	–
Specificity	70.00% (45.72%–88.11%)	–	–

AUC = Area under the curve; CI = confidence interval; HK2 = Hexokinase 2; RF = rheumatoid factor; anti-CCP = anti-cyclic citrullinated peptide antibody; RA = Rheumatoid arthritis.

Meanwhile, the diagnostic value of RF and anti-CCP in differentiating active RA from remittent RA was also examined. However, RF and anti-CCP failed as a predictive factor in differentiating active RA from remittent RA (RF: AUC = 0.589, 95%CI: 0.443–0.733; anti-CCP: AUC = 0.618, 95%CI: 0.475–0.761) **(**Table 6**)**.

**Figure 4:**
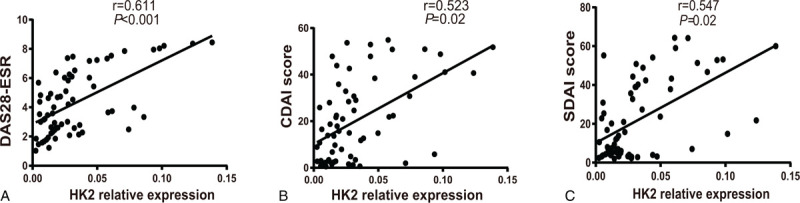
Correlations of Hexokinase 2 (HK2) expression with Disease Activity Score for 28 joints with erythrocyte sedimentation rate (DAS28-ESR) score, Clinical Disease Activity Index (CDAI) score and Simplified Disease Activity Index (SDAI) scores in Rheumatoid arthritis (RA). Spearman^'^s analysis was applied to test the correlation of HK2 expression with disease activity. *P* < .05 was considered statistically significant. HK2: Hexokinase 2; DAS28-ESR: Disease Activity Score for 28 joints with erythrocyte sedimentation rate; CDAI: Clinical Disease Activity Index; SDAI: Simplified Disease Activity Index.

Additionally, HK2 levels exhibited a positive correlation with disease activity and laboratory tests. In RA patients, HK2 correlated positively with DAS28-ESR score (*r* = 0.611, *P* < .001), CDAI score (*r* = 0.523, *P* = .02), and SDAI score (*r* = 0.547, *P* = .02) **(**Table 7, Figure 4**)**. However, the HK2 level in the PBMCs from patients with RA was not correlated with CRP, ESR, Calprotectin, RF, or anti-CCP levels; tender joint count (TJC); swollen joint count (SJC) **(**Table 7**)**.

**Table 7:** Correlation coefficients between Hexokinase 2 (HK2) levels and clinical parameters in rheumatoid arthritis patients (n = 65).

	HK2	
Variable	*r*	*P*
CRP	0.732	.07
ESR	0.653	.06
Calprotectin	0.424	.06
RF	0.543	.22
Anti-CCP	0.620	.17
TJC	0.243	.06
SJC	0.357	.07
DAS28-ESR score	0.611	<.001
CDAI score	0.523	.02
SDAI scores	0.547	.02

Values are expressed as Spearman's rank correlation coefficients. HK2 = Hexokinase 2; CRP = C-reactive protein; ESR = erythrocyte sedimentation rate; RF = rheumatoid factor; Anti-CCP = anti-cyclic citrullinated peptides; TJC = Tender joint count; SJC swollen joint count; DAS28-ESR = Disease Activity Score for 28 joints with erythrocyte sedimentation rate; CDAI = Clinical Disease Activity Index; SDAI = Simplified Disease Activity Index.

### 3.4 Correlations of HK2 status with subgroups of RA

Disease phenotypes of RA compared with the corresponding HK2 status are listed in Table 8. The prevalence of HK2 positivity was higher in moderate and high disease activity than in low disease activity and remission stage (DAS28-ESR>3.2, *P* = .02; CDAI score>10, *P* = .04; SDAI scores>11, *P* = .04). When adjusted for sex and age, bivariate logistic analysis showed that positive HK2 was independently associated with an increased risk of disease activity (DAS28-ESR>3.2 OR 4.651, 95% CI: 1.212–9.650, *P* = .02; CDAI score>10 OR 3.375, 95% CI: 1.117–9.210, *P* = .03; SDAI score >11 OR 4.316, 95% CI: 0.984–11.054, *P* = .04). Additionally, HK2 positivity was more frequently detected in the patients treated with bDMARDs than in patients treated with csDMARDs (83.33% vs 38.89%, *P* = .02). After adjusting for clinical factors, logistic analysis also identified that HK2 was independently correlated with an increased frequency of bDMARDs usage (OR 5.616, 95% CI: 1.284–16.312, *P* = .03), although the statistical power was not high due to the small number of patients treated with bDMARDs. In contrast, HK2 showed no correlation with any other variable in RA.

**Table 8:** Univariate logistic regression analyses showing the disease phenotypes of Rheumatoid arthritis (RA) in correlation to Hexokinase 2 (HK2) status as dependent variable.

			Crude			Adjusted		
Clinical Variable	n	HK2 + (%)	OR	95%CI	*P* value	OR	95%CI	*P* value
Gender								
Male	27	62.96%						
Female	38	65.79%	1.09	0.565–2.014	.71	NS		
Age								
A1<40 years	24	54.17%						
A2≥40 years	41	68.29%	1.984	0.979–4.381	.25	NS		
Disease activity								
CRP≤10 mg/L	36	55.56%						
CRP>10 mg/L	29	62.07%	1.539	0.679–3.277	.33	NS		
ESR≤40 mm/hour	48	62.50%						
ESR>40 mm/hour	17	64.71%	0.943	0.194–2.830	.26	NS		
Calprotectin≤10ug/mL	34	41.18%						
Calprotectin>10ug/mL	31	61.29%	1.873	0.925–4.871	.07	NS		
RF negativity	28	53.57%						
RF positivity	37	54.05%	2.045	0.979–3.281	.09	NS		
Anti-CCP negativity	30	56.67%						
Anti-CCP positivity	35	60.00%	1.531	0.821–3.836	.63	NS		
TJC≤3 joints	16	62.50%						
TJC>3 joints	49	67.35%	1.319	0.540–2.835	.70	NS		
SJC≤3 joints	17	52.94%						
SJC>3 joints	48	54.17%	1.261	0.450–2.988	.71	NS		
DAS28-ESR≤3.2	25	40.00%						
DAS28-ESR>3.2	40	75.00%	4.180	1.476–9.110	.02	4.651	1.212–9.650	.02
CDAI score≤10	26	46.15%						
CDAI score>10	39	74.36%	3.614	1.098–8.202	.04	3.375	1.117–9.210	.03
SDAI scores≤11	30	43.33%						
SDAI scores>11	35	71.43%	4.014	1.041–10.001	.04	4.316	0.984–11.054	.04
Medication								
csDMARDs	54	38.89%						
bDMARDs	11	81.82%	6.444	1.841–15.231	.02	5.616	1.284–16.312	.03

The date was shown with crude and adjusted odds ratios (OR) and the 95% confidence intervals (CI) accordingly. RA = Rheumatoid arthritis; HK2 = Hexokinase 2; CRP = C-reactive protein; ESR = erythrocyte sedimentation rate; RF = rheumatoid factor; Anti-CCP = anti-cyclic citrullinated peptides; TJC = Tender joint count; SJC swollen joint count; DAS28-ESR = Disease Activity Score for 28 joints with erythrocyte sedimentation rate; CDAI = Clinical Disease Activity Index; SDAI = Simplified Disease Activity Index; csDMARDs = conventional synthetic disease-modifying antirheumatic drugs; bDMARDs = biologic disease-modifying antirheumatic drugs; NS = no significant.

## 4 Discussion

The concept of metabolic reprogramming provides important information relating to pathogenesis in autoimmune diseases.^[21,22]^ OA is known as degenerative joint disease, which is characterized by articular cartilage degeneration and loss accompanied by subchondral osteosclerosis and osteophyte formation.^[23]^ A recent study reported that degeneration of cartilage may be triggered by metabolic disorders of glucose balance.^[10,24,25]^ RA is a chronic systemic autoimmune disease featured with painful joint inflammation and disability. The changes in glucose metabolism are believed to contribute to disease progression of RA.^[26,27]^ To date, recently published studies have shown that targeting the first step in glucose metabolism, HK2, could be a possible therapeutic target for RAFLSs.^[11,12]^ However, to the best of our knowledge, there are no reports regarding the assessment of HK2 levels in PBMCs from patients with RA or OA. Herein, the aim of the present study is to examine the expression and roles of HKs in the PBMCs from RA patients that may predict progression to RA.

In 2010, the American College of Rheumatology/European League Against Rheumatism (ACR/EULAR) classification criteria for RA, blood serum markers, such as CRP, ESR, ACPA (anti-carbamylated protein antibodies), RF, and anti-CCP antibodies, were adopted to diagnose RA or monitor disease activity.^[28–30]^ However, they have limited value for the diagnosis of RA due to their relatively low specificity or sensitivity. Nowadays, new laboratory markers are being explored. For example, Midkine has shown better diagnostic accuracy and detection of RA activity than traditional inflammatory markers.^[31]^ Nevertheless, the identification of more effective new biomarkers for RA is necessary.

HKs catalyse the first step of glucose metabolism and phosphorylating glucose to glucose 6-phosphate. Among all the glycolytic enzymes described to have roles in the pathogenesis of RA, HK2 may function as a superior metabolic target.^[32]^ Hence, In the present study, we focused on HK2 and validated it by real-time PCR in an RA and OA cohort.

First, the expression of HK2 was increased in PBMCs of RA and OA patients compared with that in HCs. HK2 exhibited strong clinical diagnostic value for RA (AUC = 0.808) and lower diagnostic value for OA (AUC = 0.640). Meanwhile, the diagnostic performance was detected for CRP, ESR and calprotectin in differentiating RA from HCs. The result showed that CRP, ESR and calprotectin exhibited moderate clinical diagnostic value for RA, which is consistent with the results of previous studies.^[3–5]^ However, CRP, ESR and calprotectin failed as a predictive factor in differentiating RA from OA patients, which is not found before.

Second, HK2 was significantly more upregulated in the active stage than in the remission stage of RA. The diagnostic performance supported the level of HK2 in the serum as a candidate biomarker and disease activity monitor of RA. However, RF and anti-CCP failed as a predictive factor in differentiating active RA from remittent RA. The previous study has identified that RF was not found to be independently associated with disease activity,^[33]^ which is consistent with the results of our study. Noguchi A et al identified that anti-CCP antibody significantly decreased in RA patients treated with tocilizumab,^[34]^ but our finding was not consistent with this result. Herein, a longitudinal analysis and a matched cohort study may be selected to draw more applicable conclusions.

DAS28 is the gold standard for the evaluation of disease activity, which incorporates indices including the tender joint count and swollen joint count and patient's global health evaluation based on the visual analogue scale.^[35]^ However, the tender joint count and swollen joint count in the DAS28 may reveal interrater variation. Thus, a novel biomarker with a satisfactory correlation with the disease activity assessment is needed to compensate for these defects and to evaluate treatment effects or disease development. In the present study, the serum levels of CRP, ESR, Calprotectin, RF, and anti-CCP antibodies and the DAS28-ESR, CDAI, SDAI, TJC, and SJC were first determined and analysed due to their association with the level of HK2 in PBMCs. The obtained data demonstrated that the level of HK2 was positively correlated with disease activity (DAS28-ESR, CDAI, and SDAI). However, the HK2 level in PBMCs from patients with RA was not correlated with the CRP, ESR, Calprotectin, RF, or anti-CCP antibody levels; TJC; or SJC. Moreover, logistic regression analysis revealed that HK2 was independently associated with an increased risk of disease activity in RA (DAS28-ESR>3.2; CDAI score>10; SDAI score>11). Additionally, HK2 positivity was more frequently detected in patients treated with bDMARDs than in those not treated with bDMARDs. Bustamante MF et al may have explained these results by combining overexpressed HK2 with an invasive phenotype in human FLS cell lines.^[11]^ Meanwhile, these data further indicate that HK2 may correlate with the autoimmune response and disease activity of RA, which may help clinicians to assess disease activity and guide treatment before starting a particular therapy particularly in the era of biological drugs.

Interestingly, previous studies have demonstrated a role of glucose metabolism in multiple immunocytes.^[36–38]^ Mehta MM et al identified that HK2 is dispensable for the essential function of T cell immunity.^[39,40]^ Meanwhile, HK2 is highly upregulated in activated T cells^[41,42]^ and plays important roles in B cell lymphoma cell apoptosis.^[43]^ In human monocyte-derived DCs, Toll-like receptor (TLR)-4-dependent upregulation of glycolysis leads to enhanced HK2 activity involving p38-MAPK-dependent hypoxia inducible factor-1(HIF-1) accumulation.^[44]^ Besides, phosphoinositide-3-kinase (PI3K)/AKT pathways are involved in the phosphorylation of rate-limiting mitochondrial HK2.^[45]^ Janus kinase/Signal transducer and activators of transcription (JAK/STAT) signaling was also revealed to mediate glucose uptake and HK2 expression.^[46]^ Thus, this observation together with our findings suggest that aberrant expression of HK2 may associate with pathological changes in RA by mediating related signalling pathways. More surprisingly, HK2 antagonists, including ablation of glycolytic genes or treatment with 3-bromopyruvate, significantly relieved the severity of several arthritis models.^[12,47]^ Targeting a specific intracellular compartment of HK2 (i.e., nucleus, cytosol, or mitochondria) will provide a selective way to block the harmful effect of the enzyme in RA without affecting the glucose metabolism of normal cells.^[11]^ Therefore, HK2 is an attractive and selective target for the treatment of arthritis and is safer than global glucose metabolism inhibition.

To the best of our knowledge, this is the first study to identify the relationship between HK2 expression in PBMCs and disease activity and the risk of RA. However, a number of limitations in our present study should not be neglected. First, the relatively small sample size of patients might have affected the results. Second, a more diverse disease control group and a longitudinal study is needed to draw more applicable conclusions. Third, the roles of HK2 in RA pathogenesis in the present study were not investigated. Thus, further research is urgent.

In conclusion, we verified that increased HK2 levels are a prospective candidate marker for RA diagnosis and correlate positively with disease activity in RA patients. Dysregulation of HK2 may participate in the molecular mechanism of RA and be an attractive selective metabolic target for RA treatment. The precise molecular mechanisms underlying HK2 functions in RA need further investigation.

## Author contributions

**Conceptualization:** Kai-Long Zhou.

**Data curation:** Zhen-Hua Zhu.

**Formal analysis:** Ju-Pu Zhou.

**Funding acquisition:** Bo Jiang.

**Investigation:** Ju-Pu Zhou.

**Methodology:** Kai-Long Zhou, Zhen-Hua Zhu.

**Project administration:** Jia-Ju Zhao.

**Resources:** Bo Jiang.

**Software:** Kai-Long Zhou.

**Supervision:** Kai-Long Zhou.

**Validation:** Yong Zhang.

**Writing – original draft:** Kai-Long Zhou.

**Writing – review & editing:** Kai-Long Zhou, Jia-Ju Zhao, Yong Zhang, Bo Jiang.
